# Editorial: Endocrine disruption in marine invertebrates

**DOI:** 10.3389/fendo.2022.1040939

**Published:** 2022-10-24

**Authors:** Marcos Antonio Fernandez

**Affiliations:** Marine Ecotoxicology Laboratory, Oceanography Faculty, Rio de Janeiro State University, Rio de Janeiro, Brazil

**Keywords:** marine invertebrates populations, endocrine disruption, hormonal equilibria, selective pressures, marine ecosystems structure

First, I would like to thank the Frontiers Staff, who supported every step of this Special Issue through the pandemic with a lot of care and comprehension. Second, I would like to thank the “Dream Team” of guest Editors who managed the articles revisions and greatly contributed to the work. And then, I would also thank the authors of these new articles and wish them success in their future works. For in these new times, more than ever science and technology are critical for us to understand how we are going to live in the future.

Really, we are living in a deeply changing world. Probably in no other historical moment so deep a change occurred so fast, and this change affects all aspects of our lives. The climate is changing, the ecosystems are changing, the few remaining frontiers are quickly shrinking for us in many ways. This applies for all technical, social, and economic aspects of our modern societies. Never so much information was made available for so many people, and never so much disinformation has been deliberately produced to confound and delude public opinion in various crucial aspects of our common lives.

So, what a very technical, complicate and in many instances almost incomprehensible field of knowledge such as endocrine disruption has to do within all this mess?

To begin with, the very production of scientific knowledge in a time like this is a demonstration of confidence in science as a way of looking the natural phenomena and processes, to better understand how this world of ours works, and how we can better keep it working for the benefit of the future generations. We have no B plan; we have no other spaceship that can hold all people and things we love and care for. In a time of negacionism, the very act of producing science and expanding knowledge and comprehension is an act of resistance.

And then, we came to the specific research field of endocrine disruption proper. In some instances, this kind of phenomena has been shown to produce populations losses for several species of marine invertebrates. As studies progressed, it has been shown that this kind of interference with natural hormone equilibria in different animal taxa could occur, and is occurring now, the same way as it is occurring in human populations (OMS, 2013). However, this is a very difficult, tricky research field, as a lot of understanding of the hormonal equilibria in wild animals’ populations is still missing. And, in a very human way, it is always easier for us to drive our attention to what we can see and feel, and not to things we barely know and understand.

When put together, enough evidence is currently available to indicate that among other kinds of pollutants the occurrence in the environment of compounds that interfere with the hormonal equilibria could affect wild populations of invertebrates to an extension that we still cannot properly evaluate (Fernandez) Endocrine disruption could have subtle effects, thus acting silently and almost unnoticed as a part of a set of environmental stressors that as a whole function as an artificial selection upon which natural selection will act, silently wiping out the most sensitive species of the affected areas. The result is a reduction in biodiversity, an impoverished ecosystem and a less productive area. Only detailed ecological studies can indicate the extension of this damage and more often than not, a single compound, or compound class, cannot be indicated as guilty and a target for environmental controls. We are dealing in almost all instances with mixtures of pollutants, including endocrine disruptors, of different relative potency, and affecting species with different sensitivities. So, there is still a lot of work waiting for us all around this planet. But now we have some new tools: we are more connected, we can use more information at the same time, we have incredibly powerful databases and algorithms. It is time to use them fully for ecotoxicology and integrated environmental modelling.

Let’s try to work more closely, let’s have more cooperation instead of competition, let’s look more out of our windows and under our waters. We are a very strange race, we humans. The greater the challenge, the greater the determination and the effort. Not all times, surely. But in so many instances we did the right things. So, let’s go again.

Well, it’s time to stop talking and getting back to work. A cordial salute to all people working in this important and poorly known research field.

## Author contributions

The author confirms being the sole contributor of this work and has approved it for publication.

## Acknowledgments

To Jörg Oehlmann, Toshihiro Horiguchi, Alex Ford and Peter Matthiessen, for their impressive scientific works in this research field, their kindness, and friendship; to my graduate and undergraduate students in the sea, in the lab, and at room 4034E and our long, animated discussions through the years; and to the late Carl Sagan, who better than anyone showed us how science could enlighten every day of our lives.

## Conflict of interest

The authors declares that the research was conducted in the absence of any commercial or financial relationships that could be construed as a potential conflict of interest.

## Publisher’s note

All claims expressed in this article are solely those of the authors and do not necessarily represent those of their affiliated organizations, or those of the publisher, the editors and the reviewers. Any product that may be evaluated in this article, or claim that may be made by its manufacturer, is not guaranteed or endorsed by the publisher.
